# Improving the management of behaviour that challenges associated with dementia in care homes: protocol for pharmacy–health psychology intervention feasibility study

**DOI:** 10.1136/bmjopen-2015-010279

**Published:** 2016-03-23

**Authors:** Ian D Maidment, Rachel L Shaw, Kirsty Killick, Sarah Damery, Andrea Hilton, Jane Wilcock, Nigel Barnes, Graeme Brown, Sarah Gillespie, Chris Fox, Garry Barton, Steve Iliffe, Nichola Seare

**Affiliations:** 1 Department of Clinical Pharmacy, School of Life and Health Sciences, Medicines and Devices in Ageing Cluster Lead, Aston Research Centre for Healthy Ageing (ARCHA), Aston University, Birmingham, UK; 2 Department of Psychology, School of Life and Health Sciences, Aston University, Birmingham, UK; 3 School of Life and Health Sciences, Aston University, Birmingham, UK; 4 Social Dimensions of Health Institute, University of Dundee, Dundee, UK; 5 Institute of Applied Health Research, College of Medical and Dental Sciences, University of Birmingham, Birmingham; 6 Faculty of Health and Social Care, University of Hull, Hull, UK; 7 Research Department of Primary Care & Population Health, University College London, London, UK; 8 Pharmacy, Birmingham and Solihull Mental Health NHS Foundation Trust, Birmingham, UK; 9 Department of Clinical Healthcare, Faculty of Health and Life Sciences, Oxford Brookes University, Oxford, UK; 10 Department of Clinical Psychiatry, Medical School, University of East Anglia, Norwich Research Park, Norwich, UK; 11 Norwich Medical School and Norwich Clinical Trials Unit, University of East Anglia, Norwich Research Park, Norwich, UK; 12 Research Department of Primary Care & Population Health, University College London, London, UK; 13 Aston Health Research and Innovation Cluster, Aston University, Birmingham, UK

**Keywords:** MENTAL HEALTH

## Abstract

**Introduction:**

The inappropriate use of antipsychotics in people with dementia for behaviour that challenges is associated with an estimated 1800 deaths annually. However, solely focusing on antipsychotics may transfer prescribing to other equally dangerous psychotropics. Little is known about the role of pharmacists in the management of psychotropics used to treat behaviours that challenge. This research aims to determine whether it is feasible to implement and measure the effectiveness of a combined pharmacy–health psychology intervention incorporating a medication review and staff training package to limit the prescription of psychotropics to manage behaviour that challenges in care home residents with dementia.

**Methods/analysis:**

6 care homes within the West Midlands will be recruited. People with dementia receiving medication for behaviour that challenges, or their personal consultee, will be approached regarding participation. Medication used to treat behaviour that challenges will be reviewed by the pharmacist, in collaboration with the general practitioner (GP), person with dementia and carer. The behavioural intervention consists of a training package for care home staff and GPs promoting person-centred care and treating behaviours that challenge as an expression of unmet need. The primary outcome measure is the Neuropsychiatric Inventory-Nursing Home version (NPI-NH). Other outcomes include quality of life (EQ-5D and DEMQoL), cognition (sMMSE), health economic (CSRI) and prescribed medication including whether recommendations were implemented. Outcome data will be collected at 6 weeks, and 3 and 6 months. Pretraining and post-training interviews will explore stakeholders’ expectations and experiences of the intervention. Data will be used to estimate the sample size for a definitive study.

**Ethics/dissemination:**

The project has received a favourable opinion from the East Midlands REC (15/EM/3014). If potential participants lack capacity, a personal consultee will be consulted regarding participation in line with the Mental Capacity Act. Results will be published in peer-reviewed journals and presented at conferences.

**Strengths and limitations of this study**

- This is the first study to assess the feasibility of an interdisciplinary intervention involving a medication review and a behavioural intervention.
- The study uses a mixed-methods approach and will also collect health economic data.
- The feasibility study is conducted in a single location; a future planned cluster randomised controlled trial (RCT) will be extended to cover other areas.

## Introduction

Numerous reports have highlighted that care home residents with dementia receive substandard care with a key concern being the appropriate treatment of behaviour that challenges.^1^ ^2^ Behaviour that challenges is defined as ‘any behaviour considered antisocial within the care environment or deemed dangerous to the person with dementia, their fellow residents, and staff’.^3^ Behaviour that challenges is used interchangeably with the label, behavioural and psychological symptoms of dementia (BPSD), and such behaviours occur in approximately 70% of residents and are traditionally treated with psychotropics, particularly antipsychotics.^2^ ^4^ ^5^

The number of people with dementia prescribed psychotropic medication is unknown. The National Dementia Strategy estimated that 180 000 people with dementia receive antipsychotics, yet only 20% of those prescribed are likely to benefit and the usage of such antipsychotics is implicated in the death of 1800 people with dementia annually.^2^ ^6^ The Department of Health targeted a two-thirds reduction in such usage in 2009.^2^ ^6^ However, simply targeting antipsychotics may increase the prescribing of other psychotropics such as lorazepam, which are generally ineffective and associated with serious side effects including pneumonia, fractures, falls and confusion.^4^ ^7–22^ Lorazepam usage was not included in the 2012 national audit of the treatment of behaviour that challenges (BPSD).^23^ Thus, in line with recommendations from a recent Cochrane review, future research should test interventions to limit the use of all psychotropics and not just focus on antipsychotics.^24^

The National Dementia Strategy and guidance from the Royal Pharmaceutical Society highlighted the role of pharmacist-led medication review in ensuring the appropriate treatment of BPSD.^2^ ^6^ ^25^ Successful pharmacy approaches to medication review have adopted a collaborative approach and used highly skilled clinical pharmacists; therefore, any effective intervention is likely to require the input of specialists, working collaboratively with general practitioners (GPs), community pharmacists, patients and carers.^26–32^ While recent White Papers highlight an outreach role for specialist pharmacists, there is a lack of data on the efficacy and cost-effectiveness of such an approach.^1^ ^24^ ^33–35^ Furthermore, the likely impact of a specialist medication review is unknown. Reduced psychotropics may result in people with dementia exhibiting more behaviour that challenges, thus requiring changes in their care. As a result, this study proposes to combine the pharmacy-based medication review with a health psychology-informed training package for care staff and GPs. This study will determine the fit to context, the acceptability and feasibility of a combined pharmacy–health psychology intervention to reduce psychotropics while managing behaviours that challenge effectively.

This research aims to determine whether it is feasible to implement and measure the effectiveness of a combined pharmacy–health psychology intervention incorporating a medication review and staff training package to limit the prescription of psychotropics to manage behaviour that challenges in residential and nursing home residents.

The objectives are:

1. To deliver and evaluate a full medication review to reduce the prescription of psychotropics for the treatment of behaviour that challenges.
2. To deliver and evaluate a training package for care home staff and GPs to provide appropriate care for people with dementia exhibiting behaviour that challenges.
3. To test the feasibility of proposed methods including identification of participants, recruitment and retention rates (homes and participants), acceptability and fidelity of the intervention, sample size, follow-up rates and outcome measurement tools.
4. To inform the development of a definitive cluster randomised controlled trial (CRCT).

## Methods and analysis

Details of the methods are described below (informed by SPIRIT^36^ and TIDieR^37^ guidance). The full protocol is available on the project website (http://www.aston.ac.uk/medrev/).

### Participants and recruitment

We aim to recruit three residential care homes and three nursing homes. Recruitment will be informed by Enabling Research in Care Homes (ENRICH).^38^ All care homes that include residents with dementia in the West Midlands (within 6 miles of Birmingham) with at least 40 residents will be identified from the Care Quality Commission (CQC) website and invited to participate via a letter from the chief investigator. Expressions of interest will be followed up in face-to-face meetings by the chief investigator and project manager.

On the basis of the National Dementia Strategy and previous work, we expect at least 30% of residents to meet the inclusion criteria: giving 18+ potential participants per home.^2^ ^6^ ^39^ ^40^ Recruitment and attrition rates will be monitored to inform the recruitment strategy and sample size for the CRCT; the target number of participants for this feasibility study is 45.

#### Participant inclusion criteria

1. Receiving medication (including, but not limited to, medicines in British National Formulary (BNF) sections 4.1/4.2/4.3/4.11) to treat behaviour that challenges.
2. Resident within a long-term care facility.
3. Registered with a West Midlands GP (who has also agreed to participate).
4. Dementia confirmed (dementia register, documentation of relevant read codes, confirmation of diagnosis via communication from old age psychiatry, memory clinic or clinical psychologist).
5. Patient, or personal consultee, willing to provide consent/assent.
6. A proxy informant (key worker or staff member with close working relationship) who can clearly communicate in English available.

#### Participant exclusion criteria

1. Patient, or personal consultee, unable or unwilling to provide consent or lacks necessary English-language skills.
2. On palliative care register, or has pathology requiring complex specialist medication.
3. Risk of harm in line with Alzheimer's Society guidelines (guidelines published in 2011, currently being updated and therefore not available).
4. Severe mental illness (eg, schizophrenia) where psychotropic treatment should be continued.

### Study design

This feasibility study is set within the Medical Research Council (MRC) framework for developing complex interventions.^41^ The behavourial intervention will be delivered first so that care staff have an understanding of challenging behaviour potentially as communication of an unmet need in advance of any changes in medication.

#### Health psychology-informed behavioural intervention

The health psychology-informed component of the intervention constitutes a behavioural intervention to be delivered in the form of a training package (table 1). The development of this training package has been informed by elicitation research involving a review of the evidence base and good practice guidelines for managing behaviour that challenges, and in-depth interviews with stakeholder groups. It has been developed using the Capability, Opportunity, Motivation and Behaviour (COM-B) model for the development of behavioural interventions in healthcare.^42^ This model helped to isolate the target behaviours and identify techniques for changing those behaviours (a publication detailing the development of the health psychology-informed behavioural intervention is in progress). We will collect data on the proportion of staff attending training sessions at each home. Basic demographic information on those receiving the intervention (care staff and GPs) will also be collected.

**Table 1:** Outline of training packages

*Training package for care home staff*: The training package will provide care home staff with the knowledge to understand that behaviours that challenge may be an expression of unmet need.	
Within this, the intervention aims to provide care home staff with the skills and resources to	Investigate what the unmet need might be Get to know the person with dementia as an individual to help manage their behaviour Think creatively about how to prevent challenging behaviours by making sure individuals’ needs are met Understand that behaviours that challenge are not ‘bad behaviour’.
Mode of delivery	The intervention will be delivered in brief face-to-face training programmes for all care home staff, including managers, at the care home. The training will be delivered at a time convenient for the care staff.
Content	Up to 2–3 h training package including handouts, worksheets, aide-mémoires (for handover, in residents’ rooms, communal areas), scenario-based role plays and interactive activities. Manuals will be provided to be kept in the care home for staff access.
Facilitation	The intervention training will be delivered by specialist health psychologists on the project team with support from community psychiatric nurses (CPNs) within the participating trust. Champions within homes will be identified to help maintain the key message (challenging behaviour may be an expression of unmet need).
Monitoring and evaluation	The researcher will be in regular contact with the care home managers and champions and will carry out regular visits. This will ensure staff are maintaining the key lessons of the intervention and that all materials (manuals, aide-mémoires, etc are still readily available on site) are used throughout the study period. The researcher will use field notes to support the evaluation of the intervention.
*Training package for general practitioners (GPs)*	
GPs will be given access to the care home staff training package. In addition, a short training session (face-to-face or online) will be provided which will include additional information about specific drugs, potential side effects, interactions between antipsychotics and between antipsychotics and medications for other long-term conditions, as well as information relating to advocacy skills and phrases to help GPs convey the key messages of the intervention to care home staff and families.	

#### Pharmacy-based medication review

The protocol for the medication review has been developed and has undergone initial piloting.^39^ ^40^ An experienced specialist dementia care clinical pharmacist will conduct the medication review. Prior to the review, the pharmacist will discuss the patients’ medication history, rationale for the current regimen and any potential issues with the GP and, if necessary, other clinical specialists such as community psychiatric nurses (CPNs). The review will proceed in line with Alzheimer's Society guidelines (guidelines published in 2011, currently being updated and therefore not available). It will be a process involving dialogue between the pharmacist, GP, carer and patient (with active involvement where possible; table 2 for outline).

**Table 2:** Outline of the pharmacy-based medication review

1	The primary focus of the intervention is to review psychotropics for behaviours that challenge; the pharmacist will also review all medication as per routine clinical care.
2	Establish a therapeutic alliance with the person with dementia and/or personal consultee.
3	Consult clinical records for existing diseases and current medications.
4	Collect information about the patient which may affect their disease progression or treatment.
5	Collect information about any challenging behaviour exhibited by the person with dementia.
6	Assess whether any other current medication may contribute to the challenging behaviour.
7	Review the prescription of medication including treatments used to treat psychotropic-induced adverse events.
8	Provide a verbal summary and written record of the medication review, including recommendations for medication and managing adverse events, to care staff, GP, person with dementia/personal consultee and dispensing community pharmacy, as appropriate.

#### Outcome measures

##### Primary

The Neuropsychiatric Inventory (NPI) is a caregiver-administered questionnaire that assesses 12 key neuropsychiatric symptoms.^43^ A specific nursing home version (Neuropsychiatric Inventory-Nursing Home version (NPI-NH)) designed for interviewing professional care staff will be used.^44^ ^45^ The decrease in the NPI-NH change score between baseline and 3 months will be the primary measure; it will also be collected at 6 weeks and 6 months. A decrease in four points, or more, will be considered a clinically meaningful change in the NPI-NH.^44^

##### Secondary

Collected at baseline (before the medication review), 6 weeks and 3 and 6 months (unless stated):

1. Quality of Life (QoL), DEMQoL and EuroQoL EQ-5D (proxy versions).^46–48^ Participants may not be able to respond and to reduce any ‘proxy effect’ caregivers will be requested to respond ‘as if’ they were the person with dementia.^46^ (EQ-5D only collected at baseline, 3 and 6 months; DEMQoL collected at all time points.)
2. Cognitive test: standardised Mini-Mental State Examination (sMMSE).^49^ ^50^ The sMMSE is based on original Mini-Mental State Examination (MMSE), the most widely used cognitive test worldwide with good psychometric properties.^51^ ^52^ The sMMSE provides explicit guidelines for administration and scoring to improve reliability. Both the MMSE and the sMMSE have been shown to be sensitive to the effects of medication on cognition in previous trials including a trial led by a coapplicant (CF) in the same population.^53–55^ (sMMSE only collected at baseline, 6 weeks and 3 months.)
3. All prescribed medication.
4. Costs: a modified version of the Client Service Receipt Inventory (CSRI) will be used to monitor levels of resource use associated with the medication review (including preparation and recommendations) and the use of other National Health Service (NHS) and Personal Social Service (PSS) resource items—for example, medication utilisation, GP services and hospital visits/admissions.^56^ ^57^ This will be completed by proxy. (Only collected at baseline, 3 and 6 months).

### Qualitative evaluation

The qualitative evaluation of the combined pharmacy–health psychology intervention will have two functions: to explore the context, acceptability, fidelity of the intervention and feasibility of the medication review if tested in a definitive CRCT and to examine the change process in care homes and evaluate the quality and acceptability of the training package for care staff and GPs.

#### Preintervention and postintervention interviews

Individual semistructured interviews will be conducted with key stakeholders (care staff (n=12), home managers (n=6) and GPs (n=3)). Interviews have been chosen because they give participants the opportunity to lead the discussion,^58^ ^59^ be confident that data will be kept confidential and because questions can be tailored through active listening so that the interviewer can gain a detailed understanding of the participants’ subjective experience within their role in relation to the intervention and their specific context.

Interviews will be conducted before the medication review is implemented and before the training package is delivered. Questions will focus on perceptions of the medication review and expectations related to challenging behaviour following reductions in psychotropics. Interviews will be repeated with the same staff (or representatives) following the intervention which will explore stakeholders’ perceptions of the medication review and training package. It will examine the change process in detail.

The goal of the intervention is to support through medication review and training a staged withdrawal of psychotropics, if indicated. However, we cannot predict what will be the most important implications for individual care homes. A key objective of the interviews will be to gather perceptions and opinions from people in different positions to assess the key facilitators and barriers for residents and for care staff managing the impact of the withdrawal of psychotropics, and for management staff who need to oversee the process and ensure staff are supported to manage the process.

Opinions will be sought from GPs about the intervention and what support or systems they feel need to be put in place for it to be successful. Furthermore, the triggers for prescribing psychotropics will need to be identified; the prescription may be something which family members or carers feel is necessary, or GPs may initiate it.

### Analysis

#### Statistics

The analysis will be primarily descriptive and consider recruitment and retention of homes and individuals. The number of patients screened for eligibility within each home, the number of patients meeting study inclusion criteria and the number of patients who consent to participate will be collected. For homes, the number approached and the number consenting will be calculated together with variables characterising the homes (eg, size, type, number of staff). These data will provide information on the potential flow of participants and will be used to estimate the numbers that need to be approached in a definitive trial to meet the required sample size. Information will also be collected on the interventions implemented, the number of patients completing follow-up and the completeness of data at each time point. Demographic information and baseline data will be summarised using means/SDs and/or medians/IQRs as appropriate to assess the patients entered into the study and to allow estimation of the sample size for a definitive trial.

#### Cost-effectiveness

The main purpose is to inform how data oncosts and effects would be collected within a definitive study. We will estimate completion rates and seek to identify big cost drivers in order to inform this decision. Levels of resource use associated with the medication review (including preparation and recommendations) will be recorded by those who deliver it. Additionally, we will use a modified version of the CRSI (to be completed by proxy) to monitor other NHS and PSS resource items—for example, medication utilisation, GP services and hospital visits/admissions.^57^ Appropriate unit costs will be attached to all items of resource use, including medication costs, to estimate the mean overall cost.^60^ EQ-5D^48^ and DEMQoL^46^ ^47^ data will be used to estimate the quality-adjusted life year (QALY) gain,^48^ ^61^ both will be completed via proxy.

#### Qualitative

Interview data will be analysed using framework analysis.^58^ This is a method led by thematic analysis in the first instance which then lends itself particularly well to comparing between groups and against pre-existing evidence.^59^ Initially, thematic analysis will be conducted on each individual interview to generate a set of themes. Accounts will be collated by stakeholder group and a cross-comparison analysis undertaken for each one. A framework (or matrix) of themes by group will be created to facilitate this cross-group analysis. At this point, we will assess the themes generated inductively against reviews of literature, best practice guidance and health psychology theory and incorporate anything not already identified in the interview data. Analysis of results will therefore combine empirical data with the evidence base and health psychology theory and will be used to evaluate the appropriateness and effectiveness of the intervention—both the medication review and the training package for staff.

## Ethics and dissemination

The project has received a favourable opinion from East Midlands (Nottingham 1) Research Ethics Committee (REC; reference 15/EM/3014). The most significant ethical issue associated with this study is the inclusion of people with dementia, some of whom may not have the capacity to give informed consent. Study recruitment and delivery will be informed by ENRICH and supported by staff with training and experience of working in this setting and with the study population. We will manage the study in accordance with good clinical practice and put in place the following specific two-stage process in relation to consent.

In stage 1, a key worker, on the researcher's behalf, will give eligible residents who meet the study inclusion criteria (which includes the ability to read and understand written information) the study participant information sheet; this will also be posted to the personal consultee. Personal consultees who have not responded to the letter will be contacted after 2 weeks to follow-up the invitation to participate in the study. Residents/personal consultees who wish to consider participating in the study will then be introduced to the research team who will provide any additional information that is required.

In stage 2, if consent is obtained at stage 1, fully trained research assistants (RAs) will attempt to seek consent from residents to participate in the study. Capacity will be assessed using the Mental Capacity Act (2005)^62^ ^63^ and local NHS Trust (Birmingham and Solihull Mental Health NHS Foundation Trust) guidelines. All practical steps to maximise the individual's ability to provide informed consent will be taken. When an individual has sufficient capacity, written informed consent will be obtained. Sufficient time will be taken to explain what participation entails, including any potential hazards and/or discomfort and the participant's right to withdraw without providing any explanation for doing so.^64^ ^65^

If capacity is lacking, a personal consultee will be informed about the study and consulted for advice on whether or not the potential participant would want to participate.^62^ ^63^ The Declaration of Helsinki, which states that assent is required, and British Psychological Society guidelines will also be followed. All consenting residents and their carers will be informed that they can withdraw from the study at any time. Consent will be confirmed at each interaction with participants.

There is a potential risk of harm if psychotropic medication is withdrawn and the other support mechanisms that are put in place do not effectively manage any behaviour that challenges exhibited by participants. To counter this risk, we will exclude potential participants where a risk of harm to themselves or others has been identified; this is in line with widely used guidelines from the Alzheimer's Society. Furthermore, we are putting training in place to help care staff manage any behaviour that challenges. Finally, risk of harm will also be minimised by active involvement of the GP and care staff in the medication review, if indicated the decision to withdraw the medication being determined by the GP, and as appropriate medication will be reintroduced by the GP.

The main focus for dissemination would be after any future definitive trial. However, we plan to publish the results from this feasibility study and the embedded qualitative study in peer-reviewed international journals. Our results will also be presented at international conferences.

## Discussion

Any intervention to reduce the use of antipsychotics in people with dementia must be sustainable and not lead to an increase in the use of equally dangerous other psychotropics, such as lorazepam. We propose that a full clinical medication review by a specialist dementia care clinical pharmacist, with the GP, combined with a behavioural intervention for care staff and GPs, may be able to sustainably limit the use of all psychotropics in people with dementia. This study will establish whether it is feasible to implement and measure the effectiveness of a combined pharmacy–health psychology-informed intervention. The results from the outcome measures and qualitative evaluation will inform a definitive multicentre CRCT. It will do this by providing data on participant identification, recruitment and retention rates, acceptability of the intervention, the necessary sample size and outcome measurement tools. If we can demonstrate that the intervention is effective, this will have considerable implications for practice, in particular how to embed a change in prescribing practice for behaviour that challenges and also the way that the specialist secondary care workforce is deployed.
